# Type Six Secretion System of *Bordetella bronchiseptica* and Adaptive Immune Components Limit Intracellular Survival During Infection

**DOI:** 10.1371/journal.pone.0140743

**Published:** 2015-10-20

**Authors:** Liron Bendor, Laura S. Weyrich, Bodo Linz, Olivier Y. Rolin, Dawn L. Taylor, Laura L. Goodfield, William E. Smallridge, Mary J. Kennett, Eric T. Harvill

**Affiliations:** 1 Department of Veterinary and Biomedical Sciences, The Pennsylvania State University, University Park, Pennsylvania, United States of America; 2 Department of Veterinary and Biomedical Sciences, The Pennsylvania State University, University Park, Pennsylvania, United States of America; 3 Department of Veterinary and Biomedical Sciences, The Pennsylvania State University, University Park, Pennsylvania, United States of America; 4 Department of Veterinary and Biomedical Sciences, The Pennsylvania State University, University Park, Pennsylvania, United States of America; 5 Lee Kong Chian School of Medicine and Singapore Centre on Environmental Life Sciences Engineering, Nanyang Technological University, Singapore; Louisiana State University, UNITED STATES

## Abstract

The Type Six Secretion System (T6SS) is required for *Bordetella bronchiseptica* cytotoxicity, cytokine modulation, infection, and persistence. However, one-third of recently sequenced *Bordetella bronchiseptica* strains of the predominantly human-associated Complex IV have lost their T6SS through gene deletion or degradation. Since most human *B*. *bronchiseptica* infections occur in immunocompromised patients, we determine here whether loss of Type Six Secretion is beneficial to *B*. *bronchiseptica* during infection of immunocompromised mice. Infection of mice lacking adaptive immunity (Rag1^-/-^ mice) with a T6SS-deficient mutant results in a hypervirulent phenotype that is characterized by high numbers of intracellular bacteria in systemic organs. In contrast, wild-type *B*. *bronchiseptica* kill their eukaryotic cellular hosts via a T6SS-dependent mechanism that prevents survival in systemic organs. High numbers of intracellular bacteria recovered from immunodeficient mice but only low numbers from wild-type mice demonstrates that *B*. *bronchiseptica* survival in an intracellular niche is limited by B and T cell responses. Understanding the nature of intracellular survival during infection, and its effects on the generation and function of the host immune response, are important to contain and control the spread of *Bordetella*-caused disease.

## Introduction

The ability of bacteria to persist inside host cells shields them from antibodies, complement, and other-extracellular host defenses and thus represents an effective strategy for survival during infection. Many bacteria, including *Mycobacterium tuberculosis*, *Salmonella enterica* and *Francisella tularensis*, employ a variety of different intracellular survival mechanisms to achieve long-term persistence [1–3]. Until recently, *Bordetella* species were generally considered exclusively extracellular respiratory pathogens [4], but *in vitro* studies suggest that the *Bordetella* may be able to survive intracellularly [5–12]. However, whether the bacteria utilize this intracellular survival strategy during the infection process remains unclear.

The three Classical Bordetellae, *Bordetella bronchiseptica*, *B*. *pertussis*, and *B*. *parapertussis*, cause a variety of respiratory diseases ranging from asymptomatic infection to fatal pneumonia [13]. *B*. *pertussis* and *B*. *parapertussis* are the etiological agents of whooping cough in humans and are believed to have diverged independently from a *B*. *bronchiseptica*-like ancestor [14]. *B*. *bronchiseptica* infects a wide range of mammalian hosts including mice, providing a natural-host infection model that can allow molecular manipulation of both pathogen and host. *B*. *bronchiseptica* infection induces a significant Th1-type T-lymphocyte cytokine response that is characterized by high levels of IL-2, IFN-γ, and TNF-α, but low levels of IL-5 and no IL-4 [15,16], and is generally associated with an immune response to intracellular pathogens [17,18]. Furthermore, the bordetellae were shown to be able to survive intracellularly *in vitro* in epithelial cells, dendritic cells (DCs) and macrophages [6–8]. There have been reports of the recovery of *B*. *pertussis* and *B*. *bronchiseptica* from bronchiolar lavage of mice, murine nasal cavity dendritic cells, and alveolar macrophages from HIV-infected patients [19–21], suggesting that intracellular survival is a potential mechanism employed by *B*. *bronchiseptica* during infection. However, the relevance of these observations to natural infection is unclear. Defining the role of intracellular survival in *Bordetella* disease has important implications for the development of vaccines and therapeutics.

The Type Six Secretion System (T6SS), which is widely distributed amongst Gram-negative bacteria [22], has been shown to be involved in intracellular survival of several species [23]. Further, up-regulation of most T6SS genes is dependent on contact with or intracellular growth inside the host cell [24]. In fact, many bacteria persist during infection by utilizing their T6SSs for intracellular survival and replication, including *S*. *enterica* [25], *F*. *tularensis* [26], *Aeromonas hydrophila* [27], and *Yersinia pseudotuberculosis* [28]. The T6SS in *B*. *bronchiseptica* has been reported to have a function in persistence and immunomodulation during infection [29], but its contribution to intracellular survival has yet to be characterized.

Despite the important role of the T6SS during *B*. *bronchiseptica* infection, a subset of recent *B*. *bronchiseptica* isolates from the predominantly human-associated Complex IV are missing the T6SS. Since most *B*. *bronchiseptica* human infections occur in immunocompromised individuals [30–35], we aimed to determine whether loss of the T6SS might be beneficial for *B*. *bronchiseptica* during infection of immunocompromised hosts. Here we compare the wild-type *B*. *bronchiseptica* strain RB50 with RB50Δ*clpV*, an isogenic *B*. *bronchiseptica* mutant with an in-frame deletion in the *clpV* ATPase of the T6SS, during infection of mice lacking adaptive immunity (B and T cells). We show that loss of T6SS function results in a hypervirulent phenotype characterized by early host lethality of immunodeficient mice due to high numbers of predominantly intracellular bacteria in systemic organs. In contrast, wild-type *B*. *bronchiseptica* kill their cellular hosts via a T6SS-dependent mechanism and are therefore not recovered from systemic organs. A more careful examination revealed an intracellular stage in the lungs of both wild-type and immunodeficient mice, demonstrating that *B*. *bronchiseptica* can occupy an intracellular niche during natural host infection. These results reveal the ability of *B*. *bronchiseptica* to survive intracellularly and demonstrate that both the T6SS and adaptive immune components contain bacteria within the respiratory tract and limit *B*. *bronchiseptica* intracellular survival during infection.

## Materials and Methods

### Analysis of clinical strains

The T6SS locus in the genomes of 58 *B*. *bronchiseptica* strains isolated from humans (17 strains), a variety of different mammals (31 strains), turkeys (9 strains) and from an unidentified host (1) were analyzed [36–39]. All genomes were compared against the genome of strain RB50, and the presence of the T6SS locus (BB0793-BB0818) [29] in the individual genomes as well as the presence of pseudogenes was assessed visually using the Artemis Comparison Tool (ACT) [40].

### Bacterial Strains and growth

*Bordetella bronchiseptica* strain RB50 and its isogenic, in-frame deletion mutant RB50Δ*clpV* have been previously described [29,41]. RB50 and RB50Δ*clpV* were grown and maintained on Bordet-Gengou (BG, Difco) agar supplemented with 10% defibrinated sheep’s blood (Hema Resources) and 20 ug/ml streptomycin (Sigma). For infections, bacteria were grown overnight at 37°C to mid-log phase in Stainer Scholte liquid broth (SS).

### Mouse Experiments

Four- to six- week old C57Bl/6 and Rag1^-/-^ mice were ordered from Jackson laboratories (Bar Harbour, ME) and were bred in a pathogen-free facility at the Pennsylvania State Laboratory (University Park, PA). All experiments were conducted following institutional guidelines, and all animal experiments were conducted as previously described [29,42,43]. Briefly, the number of bacterial colony units in liquid SS culture was determined by the optical density measured by absorbance of light at 600 nm. The bacteria were diluted to 1x10^7^ CFU/mL in phosphate buffered saline (PBS), and inocula were confirmed by plating dilutions on BG agar and counting resultant colonies after incubation for two days at 37°C. For inoculation, mice were lightly sedated with 5% isofluorane (IsoFlo, Abbott Laboratories) and were inoculated with 5x10^5^ CFU bacteria by gently pipetting 50 μL of the inoculum onto their external nares. To quantify bacterial numbers in respiratory tract and systemic organs, mice were euthanized via CO_2_ inhalation, and the indicated organs were excised. Tissues were homogenized in one mL PBS, serially diluted and plated on BG agar containing 20 μg/mL streptomycin, and colonies were counted after incubation at 37°C for two days. Mice with lethal bordetellosis indicated by ruffled fur, hunched stature, and limited responsiveness were euthanized to prevent unnecessary suffering. For survival curves, Rag1^-/-^ mice were lightly sedated with 5% isofluorane and inoculated with 5x10^5^ CFU RB50 or RB50Δ*clpV*. Mice were monitored over the course of the experiment and any mice that were hunched over, had labored breathing, or displayed unresponsiveness or lack of motility were removed from the experiment and euthanized to prevent unnecessary suffering.

### Cytokine ELISA

Cytokine analysis was conducted as previously described [44]. Briefly, mice were inoculated with 5x10^5^ CFU RB50 or RB50Δ*clpV* and spleens and lungs were collected and homogenized on day 21 for cytokine analysis. Interleukin 1β (IL-1β), Interleukin 6 (IL-6), Interleukin 10 (IL-10), Interleukin 17 (IL-17), Tumor Necrosis Factor α (TNF-α), and Interferon Gamma (IFN-γ) concentrations were determined via ELISA according to manufacturer’s instructions (R&D Systems).

### Pathology

Twenty-one days following inoculation with RB50 or RB50Δ*clpV*, mice were euthanized and the lungs were inflated with 1.5 ml 10% formalin in PBS. Spleens were excised and placed in 10% formalin in PBS. Tissues were processed and stained with hematoxylin and eosin (H&E) at the Animal Diagnostic Laboratory at the Pennsylvania State University in University Park, PA. Tissue sections were analyzed and scored on a qualitative scale as previously described [29] by one of the authors (M.J. Kennett) who is experienced in rodent pathology and was blinded to the experimental treatments. Descriptive evaluations of lesions were recorded, and lung and spleen lesions were graded on a scale ranging from 0 to 5: A score of 0 was given for sections with no lesions and no inflammation; 1 for sections with few lesions (less than 10% tissue affected) and slight inflammation; 2 indicated mild lesions (11–20% tissue affected); 3 was given for moderate lesions (21–30% of the lung/spleen tissue affected); 4 had extensive lesions with marked inflammation (31–50% tissue affected); and a score of 5 was given for samples exhibiting extensive lesions with high inflammation (>51% lung / spleen tissue affected).

### Intracellular Survival Assay

Murine RAW 264.7 macrophages obtained from ATCC (ATCC TIB-71) were grown in Dulbecco’s modified Eagles medium (DMEM, Difco) supplemented with 10% fetal bovine serum, 1% penicillin-streptomycin, 1% nonessential amino acids, and 1% sodium pyruvate. Cells were grown to 80% confluency in 5% CO_2_ in 96-well tissue culture treated plates (Greiner Bio-One) at 37°C. Cells were primed for two hours with 1000 U/mL recombinant IFNγ in DMEM in 5% CO_2_ at 37°C, and then bacterial suspensions of RB50 or RB50Δ*clpV* were added to wells at a multiplicity of infection (MOI) of 100. Plates were centrifuged at 5000 RPM for 5 min at room temperature (RT) and were incubated at 37°C. After 1 hour, 100 μL of 0.1% triton X solution (Sigma) in PBS was administered to a subset of wells, followed by a 5 minute incubation at RT and vigorous pipetting to lyse open cells. 10 μL dilutions were serially diluted and plated on BG to quantify total bacteria (intracellular and extracellular) present after 1 hour. At one hour, supernatant was removed from remaining wells and replaced with 100 μL of 100 μg/mL gentamicin solution (Sigma-Aldrich) in DMEM to remaining sample wells. Plates were incubated in 5% CO_2_ at 37°C, and then at 1, 24, and 48 hours post-gentamicin addition, appropriate wells were washed 3x with DMEM and treated with 100 μL 0.1% triton X as described above for enumeration of intracellular bacteria. At 24 hours, the supernatants were replaced with 10 μg/mL gentamicin solution in DMEM to prevent uptake of gentamicin into intracellular compartment of RAW264.7 cells.

### Modified Intracellular Survival Assay

A modified intracellular survival assay conducted on homogenates has been previously described [19]. Briefly, C57Bl/6 and Rag1^-/-^ mice were inoculated with 5x10^5^ CFU RB50 or RB50Δ*clpV* and euthanized on day 21 post-inoculation (p.i.). Nasal cavities, lungs, spleens and livers were excised and homogenized in one mL PBS. 100 μL of the homogenate was removed and was serially diluted and plated on BG agar for quantification of total bacteria. Gentamicin (100 μg/mL) was added to remaining homogenates and samples were incubated for 1 hour at 37°C. After three washes to remove the antibiotic, remaining bacteria were resuspended in 0.1% triton X, serially diluted, and plated on BG supplemented with 20 μg/ml streptomycin for intracellular bacterial enumeration. As a control to test for gentamicin effectiveness in each organ, spleens, livers, lungs, and nasal cavities excised from naïve Rag1^-/-^ mice were homogenized and 10^7^ CFU RB50 or RB50Δ*clpV* were added to each organ. Gentamicin was shown to be 99.999% effective at killing extracellular bacteria introduced to lungs, nasal cavity, and spleen tissues, and 99% effective at killing extracellular bacteria in the liver (data not shown). Samples were incubated at 37°C for one hour, followed by washes to remove antibiotic, and the remaining viable bacteria were enumerated via serial dilution and plating on BG agar.

### Cytotoxicity Assay

Murine RAW 264.7 macrophages from ATCC were grown in DMEM supplemented with 10% fetal bovine serum, 1% penicillin-streptomycin, 1% nonessential amino acids, and 1% sodium pyruvate. Cells were grown to 80% confluency in 5% CO_2_ in 96-well tissue culture treated plates (Greiner Bio-One) at 37°C. DMEM was then replaced with RPMI medium lacking phenol red with 5% fetal bovine serum, 1% L-glutamine, 1% nonessential amino acids, and 1% sodium pyruvate two hours prior to start of the assay. RB50 or RB50Δ*clpV* were added to plates at an MOI of 100 and the plates were centrifuged at 5000 RPM for 5 min at RT and were incubated in 5% CO_2_ at 37°C. One hour later, the supernatant was replaced with 100 μg/mL gentamicin in RPMI lacking phenol red to kill extracellular bacteria and the plates were incubated once again in 5% CO_2_ at 37°C. After one hour or 24 hours, supernatants were collected from wells and lactate dehydrogenase (LDH) release was measured to quantify levels of cytotoxicity using a Cytotox96 kit (Promega) according to the manufacturer’s instructions.

### IV Injections

RB50 or RB50Δ*clpV* grown in SS to mid-log phase were re-suspended in PBS and 200 μL of 5x10^5^ CFU/mL were intravenously injected into the tail veins of mice. Mice were dissected 1 and 7 days post-injection and lungs, livers, spleens, and kidneys were homogenized in one mL PBS, serially diluted and plated on BG agar containing 20 μg/mL streptomycin, and colonies were counted after incubation at 37°C for two days. Blood was collected via retro-orbital bleed, spun down at 5000 RPM for 5 min, and serum was then serially diluted, plated, and incubated at 37°C for two days.

### Trypan Blue Exclusion Assay

RAW267.4 macrophage cells were grown in 96 well plates and infected with RB50 or RB50Δ*clpV* at an MOI of 100 and incubated at 37°C. Supernatants were replaced with 100 μL 100 μg/mL gentamicin solution after one hour, and one and twenty-four hours post-addition of gentamicin a subset of RAW264.7 cells were mixed 1:1 with trypan blue dye (Life Technologies). Total numbers of cells and the number of dead cells were enumerated for each timepoint.

### Statistical Analysis

The mean +/- standard error (error bars in figures) was determined for all appropriate data. Two tailed, unpaired student’s t tests were used to determine statistical significance between two normally distributed populations. When more than two groups were analyzed, one- and two-way analysis of variance (ANOVA) tests were used. Survival curves were generated with the Kaplan-Meier method, and Log-Rank test was used to compute significance. Graphpad Prism version 6.04 was used to conduct these statistical tests and to generate figures.

### Ethics Statement

This study was carried out in strict accordance with the recommendations in the Guide for the Care and Use of Laboratory Animals of the National Institutes of Health. The protocol was approved by the Institutional Animal Care and Use Committee at The Pennsylvania State University at University Park, PA (#46284 Bordetella-Host Interactions). All animals were anesthetized using isoflourane or euthanized using carbon dioxide inhalation to minimize animal suffering.

## Results

### 29% of Complex IV *B*. *bronchiseptica* isolates have lost their Type Six Secretion System

**Fig 1:**
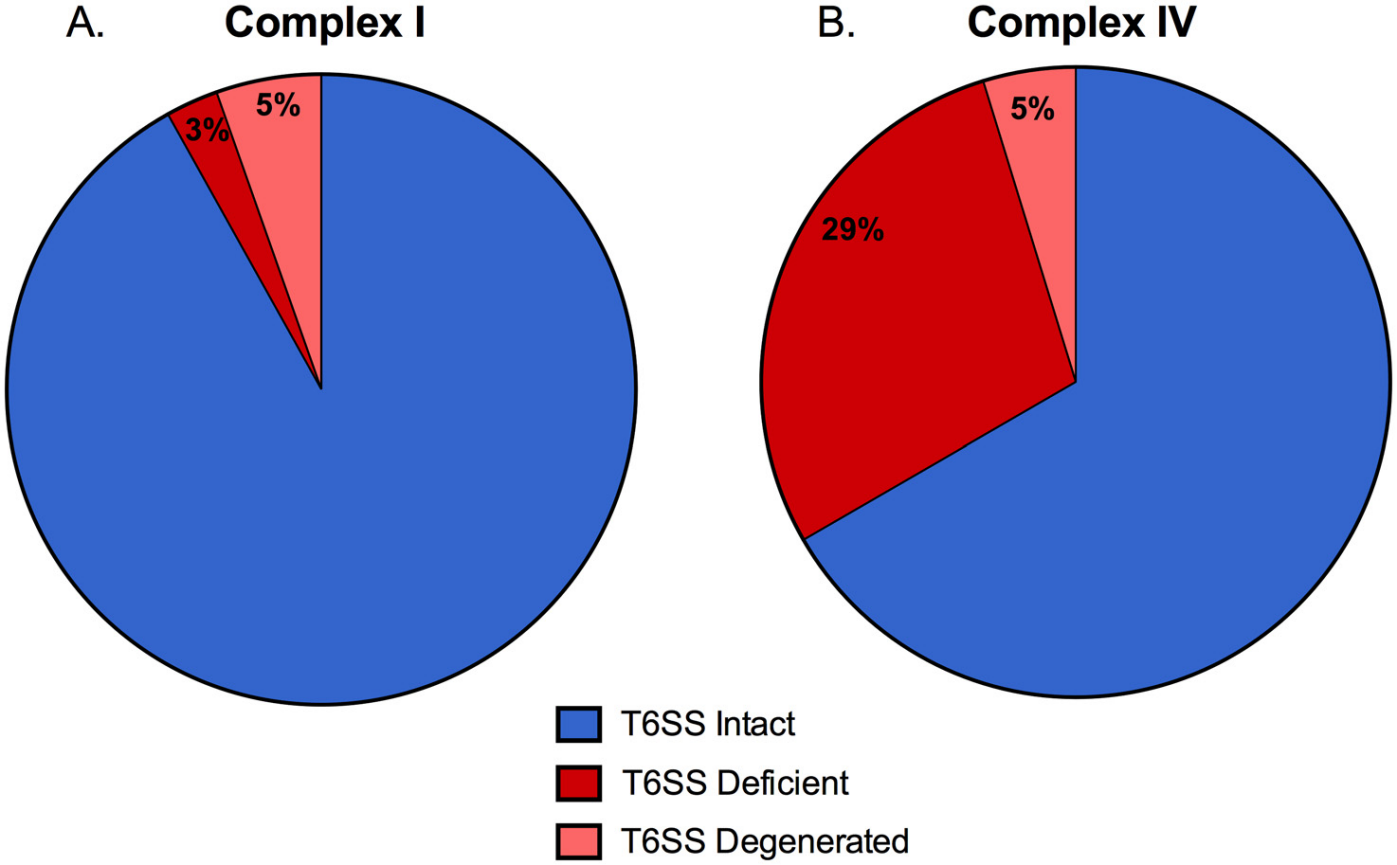
Clinical *B*. *bronchiseptica* strains that have lost their T6SS locus are aggregated in Complex IV. Genomes of 58 *B*. *bronchiseptica* clinical isolates were compared to prototypical RB50 genome. Presence of T6SS loci, as well as presence of pseudogenes in T6SS loci, was determined for all clinical isolates. Clinical strains containing an intact T6SS (blue), strains lacking a T6SS locus (red), and strains containing a pseudogene in the T6SS locus (pink) were divided based on whether they come from Complex I (A) or Complex IV (B).

The T6SS is a crucial virulence factor for *B*. *bronchiseptica* that has been shown to increase pathology and cytotoxicity, affect cytokine induction, and aid in bacterial persistence during infection of wild-type mice [29]. However, an analysis of 58 recently sequenced *B*. *bronchiseptica* isolates revealed that 7 of the total isolates (12%) have lost their entire T6SS locus. In addition, 3 isolates (5%) contain pseudogenes (referred to in this paper as T6SS-degenerate) in their T6SS locus, implying that their T6SS may be non-functional. 6 of the 7 T6SS-deficient strains isolated and one T6SS-degenerate strains are from Complex IV, which is comprised mainly of human-isolated *B*. *bronchiseptica* strains [14]. Thus, 29% of Complex IV strains are T6SS-deficient and 5% are T6SS-degenerated (Fig 1A). In contrast, only 3% of Complex I *B*. *bronchiseptica* strains analyzed (1/37) are T6SS-deficient and 5% (2/37) are T6SS-degenerated (Fig 1B). This data suggests that loss of the T6SS may be linked to *B*. *bronchiseptica* survival in the human population, and may contribute to infection of immunocompromised patients.

### Deleting the *clpV* component of T6SS results in hypervirulence in immunodeficient mice

**Fig 2:**
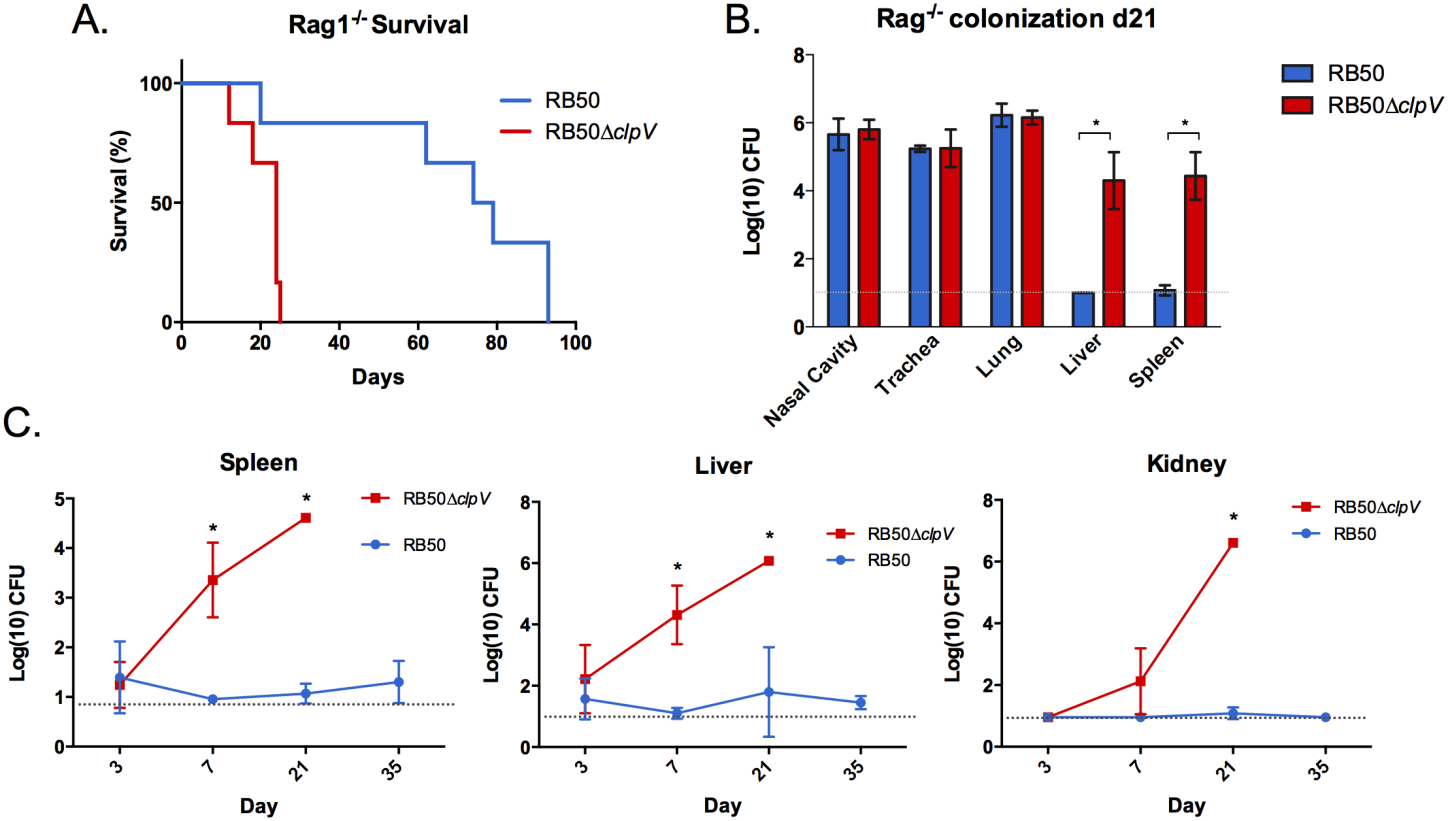
The T6SS modulates virulence and bacterial dissemination. (A) Groups of Rag1^-/-^ mice (n = 8) were inoculated with 5x10^5^ CFU of RB50 (blue) or RB50Δ*clpV* (red) and were monitored for survival. (B) Groups of Rag1^-/-^ mice (n = 4 per group) were inoculated with 5x10^5^ CFU of RB50 (blue) versus RB50Δ*clpV* (red) and dissected on day 21 p.i. for bacterial enumeration in respiratory tract and systemic organs. The experiment was performed three times with similar results and a representative data set is shown. (C) Rag^-/-^ mice were inoculated with 5x10^5^ CFU RB50 (blue) versus RB50Δ*clpV* (red) and bacteria were enumerated from the spleen, liver, and kidney on days 3, 7, 21, and 35. With the exception of RB50Δ*clpV* on day 21 (n = 1), three mice were sacrificed per group per timepoint. * denotes p value <0.05. Grey dotted line indicates the limit of detection.

Since most *B*. *bronchiseptica* human infections occur in immunocompromised patients [30–35,45,46], we hypothesized that T6SS-deficient *B*. *bronchiseptica* may be able to infect and persist in immunodeficient mice. To test this, mice lacking T cells, B cells, and antibodies (Rag1^-/-^ mice) were inoculated with RB50 or RB50Δ*clpV*, an isogenic mutant lacking the *clpV* ATPase gene of the T6SS [29]. While most Rag1^-/-^ mice infected with RB50 survived beyond day 60, those infected with RB50Δ*clpV* succumbed to lethal bordetellosis by day 24 p.i. (p<0.05)(Fig 2A). Quantification of RB50 and RB50Δ*clpV* in respiratory tract organs of Rag1^-/-^ mice on day 21 (prior to death with RB50Δ*clpV* infection) revealed similar numbers of *Bordetella* (Fig 2B) indicating that the hypervirulence of the Δ*clpV* strain is not caused by increased bacterial load in the respiratory tract. We therefore hypothesized that the early host death caused by RB50Δ*clpV* may result from systemic bacterial infection. Rag1^-/-^ mice inoculated with either RB50 or RB50Δ*clpV* were thus assessed for bacterial numbers in the respiratory tract (nasal cavity, trachea, and lungs) or systemic organs (liver and spleen) 21 days post-infection. While similar numbers of RB50 and RB50Δ*clpV* were recovered from respiratory tract organs, only RB50Δ*clpV* was recovered from systemic organs (Fig 2B). These data indicate that a functional T6SS prevents *B*. *bronchiseptica* from colonizing systemic organs of Rag1^-/-^ mice.

In order to determine how rapidly RB50Δ*clpV* reaches systemic organs, we enumerated bacterial numbers on days 3, 7, and 21. RB50Δ*clpV* was recovered from the liver and spleen of Rag1^-/-^ mice as early as day 3 p.i., and additionally from the kidneys by day 7. RB50Δ*clpV* numbers then increased in all three organs by day 21 (Fig 2C). In contrast, RB50 was only recovered from systemic organs on day 3, but was then completely absent or present in only small numbers on subsequent days (Fig 2C). A human *B*. *bronchiseptica* isolate naturally missing a T6SS locus [36] (D445) was also recovered systemically from Rag1^-/-^ mice by day 3 p.i. and on subsequent days (S1 Fig and data not shown). Importantly, both RB50 and RB50Δ*clpV* were recovered from systemic organs of immune-competent mice (wild-type C57Bl/6) on day 3 p.i., but only RB50Δ*clpV* was recovered from systemic organs by day 7 p.i., suggesting that the T6SS limits *B*. *bronchiseptica* persistence in systemic organs irrespective of host immune status (S2 Fig). Thus, a functional T6SS limits *B*. *bronchiseptica* survival and growth in systemic organs of immunodeficient and wild-type mice during infection.

### RB50Δ*clpV* systemic infection is not the result of inflammatory response

**Fig 3:**
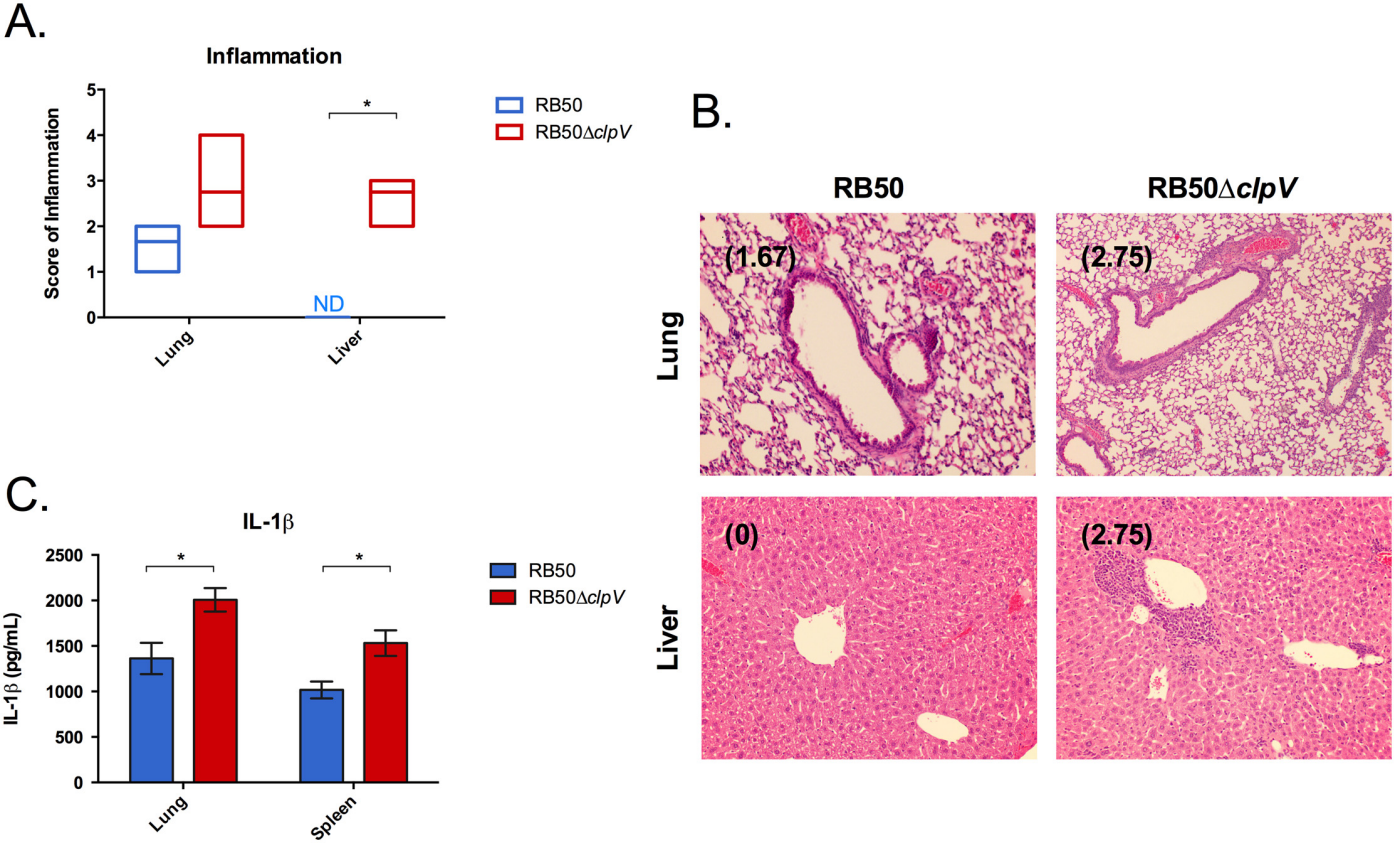
*clpV* lowers inflammation and pathology *in vivo*. (A) Groups of four Rag1^-/-^ mice were inoculated with 5x10^5^ CFU of RB50 (blue) or RB50Δ*clpV* (red) and a histopathological analysis was conducted on the lung and liver of infected mice on day 21 p.i. for scoring of inflammation. (B) Representative H&E lung and liver sections from Rag1^-/-^ mice on day 21 p.i. after inoculation with 5x10^5^ CFU of RB50 (blue) or RB50Δ*clpV* (red) with average pathology scores in parentheses. (C) Groups of Rag1^-/-^ mice were inoculated with 5x10^5^ CFU RB50 (blue) and RB50Δ*clpV* (red) and elicited IL-1β levels were determined from the lung and spleen on day 21 p.i. This experiment was performed twice with similar results and a representative dataset is shown. ND signifies not detected. * denotes p value <0.05.

We hypothesized that immunodeficient mice were dying from an overwhelming inflammatory response with infection of the mutant. To test this, we performed histological examination of livers and lungs of infected mice. Rag1^-/-^ mice infected with RB50Δ*clpV* exhibited higher levels of inflammation in the liver than those infected with RB50, characterized by primarily perivascular neutrophilic infiltrates with foci of necrosis (p<0.05)(Fig 3A). Despite similar levels of colonization in the lungs at this timepoint (Fig 1B), infection with RB50Δ*clpV* resulted in a trend toward higher inflammatory cell infiltration inflammation and necrosis in the lungs than infection with RB50 (Fig 3A and data not shown). Also, while RB50 caused no significant lesions in the liver, RB50Δ*clpV* caused high hepatic inflammation with primarily perivascular mixed inflammatory infiltrates by day 21 (Fig 3B).

To determine whether the T6SS modulates local and systemic cytokine production, cytokine levels were measured in the lungs and spleens of Rag1^-/-^ mice inoculated with RB50 or RB50Δ*clpV*. Wild-type RB50 induced lower levels of IL-1β than RB50Δ*clpV* in the lungs and spleens of Rag1^-/-^ mice by day 21, indicating that the T6SS suppresses IL-1β production during infection (*p*<0.05)(Fig 3C). Together, these data suggest that the *B*. *bronchiseptica* T6SS modulates innate immunity by lowering pathology, cell recruitment, and IL-1β production both locally and systemically in Rag1^-/-^ mice.

**Fig 4:**
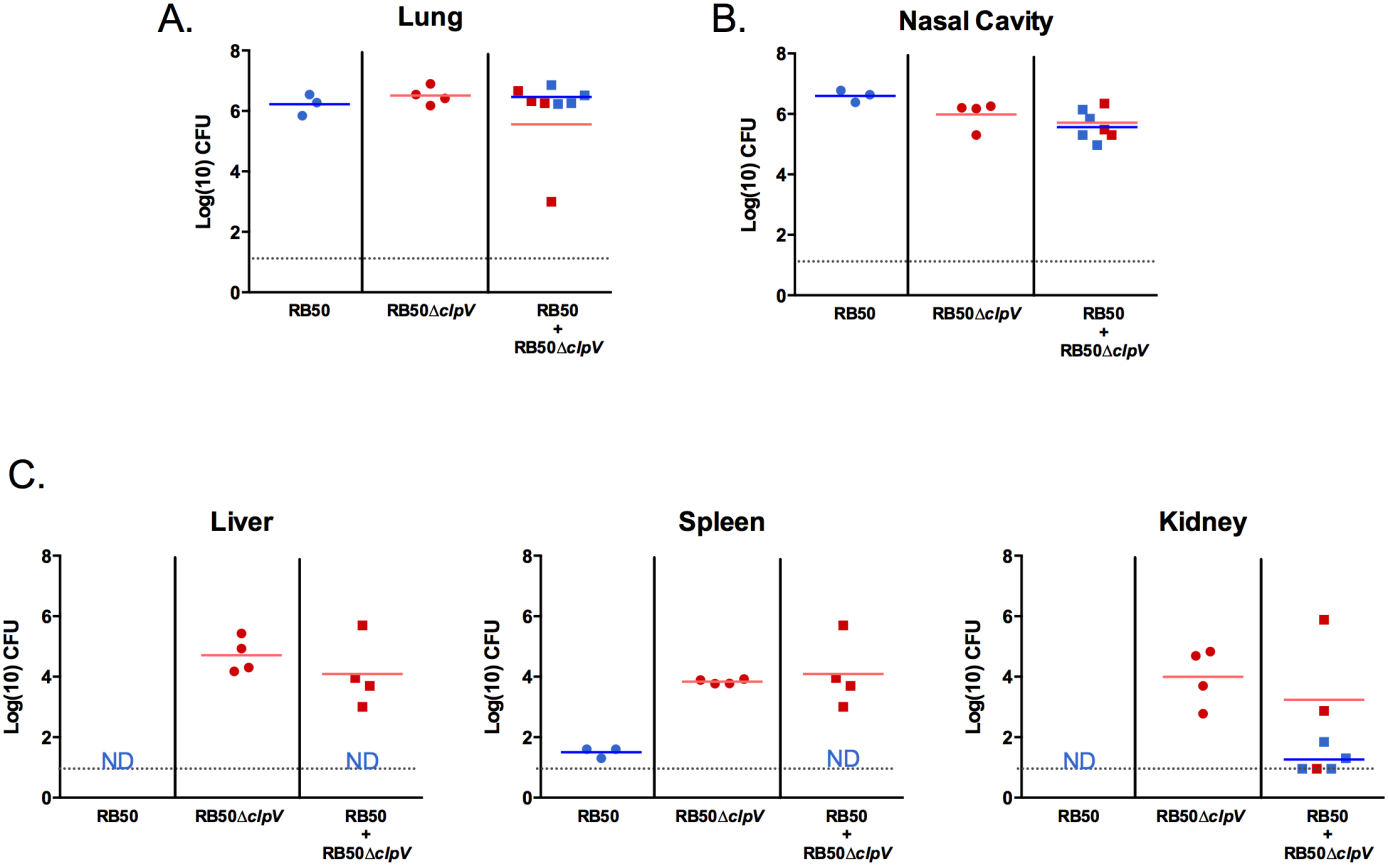
Loss of *clpV* is required for systemic recovery. Groups of Rag1^-/-^ mice were either infected singly with RB50 (blue circles, n = 3) or RB50Δ*clpV* (red circles, n = 4) or co-infected with RB50 and RB50Δ*clpV* (red squares and blue squares, n = 4), and (A) respiratory tract bacterial numbers or (B) systemic organ bacterial numbers were enumerated on day 21 post-inoculation. This experiment was performed twice with similar results and a representative dataset is shown. ND signifies not detected. Grey line indicates the limit of detection.

We hypothesized that spread and subsequent survival of RB50Δ*clpV* in systemic organs could be due to the different immune response induced by the mutant, much like *S*. *aureus* lipoprotein mutants generate a variant host response culminating in dissemination and lethal infection [47]. If this were the case, then co-inoculation with RB50 would provide T6SS-mediated immune modulation that would affect the fate of RB50Δ*clpV*. Alternatively, if systemic survival is intrinsic to the bacteria harboring the mutation and not mediated by indirect effects on the host, then the T6SS-dependent immune response generated by co-infection would not alter RB50Δ*clpV* systemic infection. To distinguish between these possibilities, Rag1^-/-^ mice were inoculated with RB50 alone, RB50Δ*clpV* alone, or co-inoculated with RB50 and RB50Δ*clpV*. Similar numbers of RB50 and RB50Δ*clpV* were recovered from respiratory tract organs (lungs and nasal cavities) of Rag1^-/-^ mice by day 21 p.i., regardless of whether they were inoculated separately or co-inoculated (Fig 4A). However, only RB50Δ*clpV* was recovered from systemic organs (*p*<0.05), and the numbers of RB50Δ*clpV* recovered from co-inoculated mice were similar to RB50Δ*clpV* inoculated alone (Fig 4B). Therefore, a functional T6SS provided by RB50 in the co-inoculation did not hinder the ability of RB50Δ*clpV* to reach and remain in systemic organs by day 21 p.i., and conversely the altered immune response with RB50Δ*clpV* infection still did not enable systemic survival of RB50 in the co-infected mice (Fig 4B). Loss of *clpV* only affects the bacteria with the mutation, suggesting that the T6SS affects direct interactions between bacteria and immune cells recruited to the site of infection. Additionally, numbers of RB50 and RB50Δ*clpV* were efficiently lowered in systemic organs when injected directly into the bloodstream (S3 Fig), once again suggesting that the intracellular niche may be utilized to protect *B*. *bronchiseptica* and enable trafficking to systemic organs.

### Deletion of *clpV* increases *B*. *bronchiseptica* intracellular survival *in vitro*

**Fig 5:**
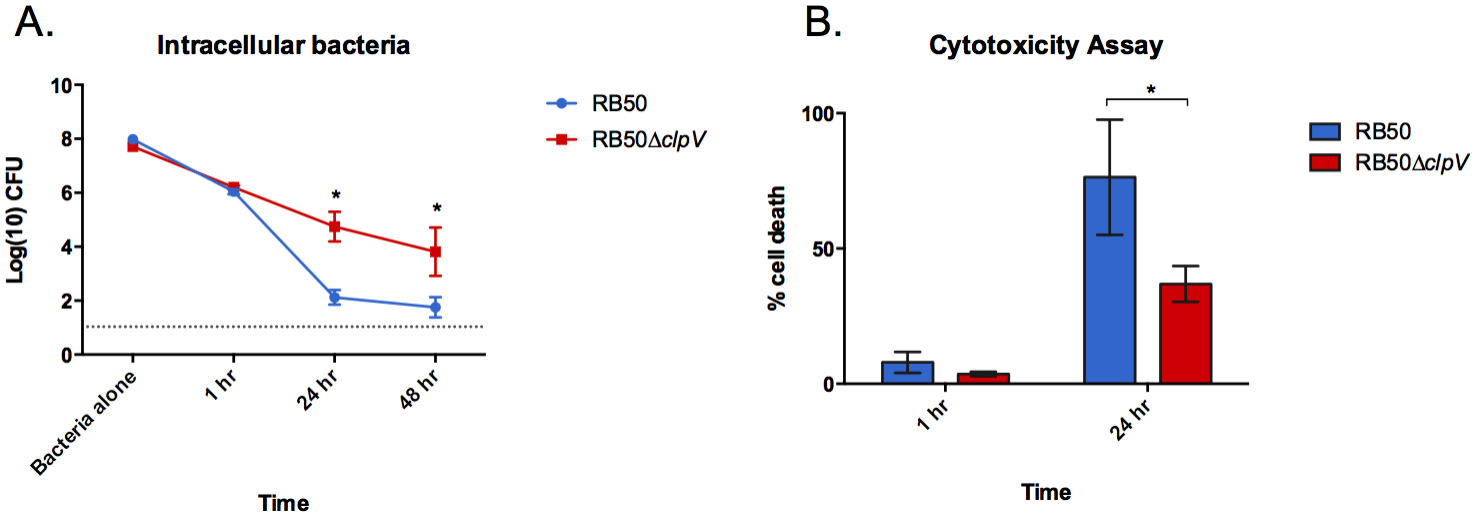
Deletion of *clpV* increases intracellular survival *in vitro*. (A) RAW264.7 macrophages were infected with RB50 (blue, n = 4) or RB50Δ*clpV* (red, n = 4) at an MOI of 100 and bacterial invasion and intracellular survival was determined at 1, 24, and 48 hour after addition of gentamicin. The experiment was conducted five times with similar results and a representative dataset is shown. (B) The cytotoxicity of RAW264.7 macrophages infected with RB50 (blue) or RB50Δ*clpV* (red) at an MOI of 100 was determined 1 hour and 24 hours after gentamicin application. The average percent cytotoxicity of four wells in three different experiments was measured by (LDH release from a well / LDH release from positive control well) x 100 ±SE is shown. * denotes p value <0.05. Grey line indicates limit of detection.

Based on the results above, we hypothesized that *B*. *bronchiseptica* manipulates Antigen Presenting Cells (APCs) to house and traffic bacteria to systemic organs during infection, and that long-term survival in those immune cells is achieved through the loss of the T6SS. To determine whether loss of the T6SS affects intracellular survival, a gentamicin protection assay was performed to estimate intracellular survival of RB50 and RB50Δ*clpV* in RAW264.7 macrophages [48]. After a 1 hour treatment with gentamicin, similar numbers of RB50 and RB50Δ*clpV* were recovered from RAW264.7 macrophages (Fig 5A) suggesting that phagocytosis and early intracellular survival of *B*. *bronchiseptica* is T6SS-independent. However, after 24 hours numbers of intracellular RB50Δ*clpV* (~1x10^5^ CFU/mL) were approximately 1000-fold higher than that of RB50 (~1x10^2^ CFU/mL) (*p*<0.05) and remained over 1000-fold higher at 48 hours (p<0.05)(Fig 5A). Similarly, infection of RAW264.7 macrophages with a human isolate naturally lacking its T6SS (D445) yielded similarly high levels of recovered intracellular bacteria by 24 hours (S4 fig). Thus, loss of a functional T6SS increased *in vitro* intracellular survival of *B*. *bronchiseptica* in RAW264.7 macrophages. A cytotoxicity assay [29] was performed to analyze whether lower intracellular RB50 recovery by 24 hours correlates with higher cell death. Intracellular RB50 and RB50Δ*clpV* caused similar levels of cell death within 1 hour (Fig 5B), but by 24 hours intracellular RB50 caused higher levels of cell death than RB50Δ*clpV* (*p*<0.05)(Fig 5B). A trypan blue exclusion cell viability assay [49] confirmed these results (data not shown). Therefore, *B*. *bronchiseptica* can survive intracellularly in RAW264.7 macrophages, but the T6SS inhibits long-term intracellular survival by killing its eukaryotic host cell. These data suggest that APCs may act as vehicles for the transport of *B*. *bronchiseptica* to systemic organs during *in vivo* infection and may enable persistence of RB50Δ*clpV* in systemic organs of Rag1^-/-^ mice.

### The T6SS and adaptive immune components limit *in vivo* intracellular survival

**Fig 6:**
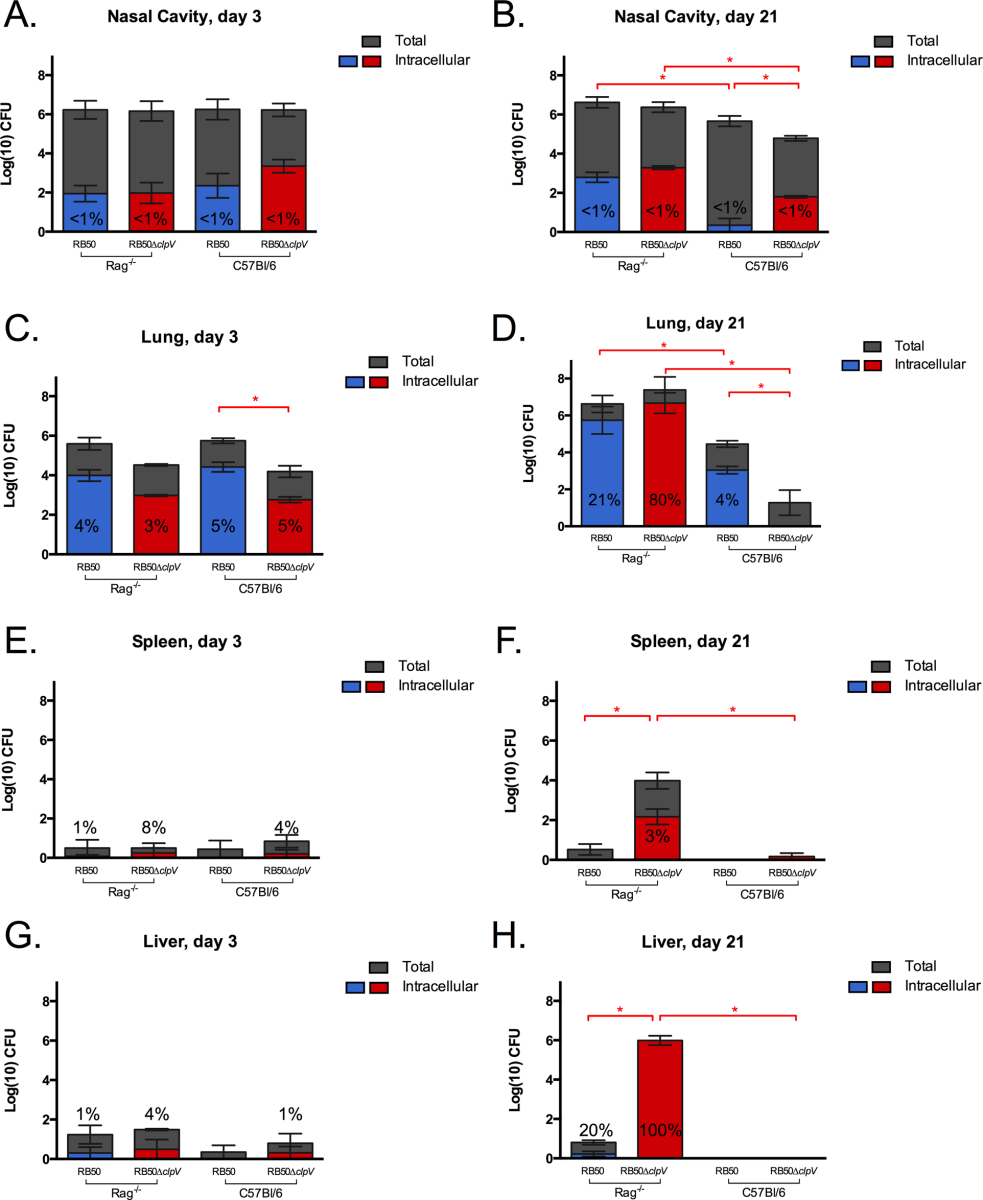
Deletion of *clpV* increases intracellular survival *in vivo*. Groups (n = 3) of Rag1^-/-^ and C57Bl/6 mice were inoculated with 5x10^5^ CFU RB50 or RB50Δ*clpV* and total bacterial numbers (grey) and intracellular RB50 (blue) and RB50Δ*clpV* (red) numbers were enumerated from the nasal cavity (A, B), lungs (C, D), spleen (E, F), and liver (G, H) early (day 3) and late (day 21) post-infection. This experiment was conducted twice with similar results and a representative dataset is shown. Red * denotes p value <0.05 for comparison of intracellular bacterial populations.

To investigate whether the T6SS affects intracellular survival *in vivo*, we gentamicin treated homogenized organs recovered from mice inoculated with RB50 or RB50Δ*clpV* and then enumerated surviving (intracellular) bacteria [19]. Less than 1% of RB50 and RB50Δ*clpV* recovered from the nasal cavities of both C57Bl/6 and Rag1^-/-^ mice on days 3 and 21 p.i. survived intracellularly (Fig 6A and 6B). However, approximately 4% of RB50 in the lungs of C57Bl/6 mice on days 3 and 21 p.i. were gentamicin resistant (Fig 6C and 6D), suggesting that a proportion of *B*. *bronchiseptica* survives intracellularly in the lungs during infection and that there may be organ-specific differences in the proportion of bacteria that are intracellular. A similar proportion of RB50 (5%) was intracellular in lungs of Rag1^-/-^ mice on day 3 p.i., but those numbers increased to 21% by day 21 p.i. (Fig 6C and 6D). Intracellular *B*. *bronchiseptica* was therefore observed in both wild-type and immunodeficient mice but increased over time only in the latter, indicating that adaptive immune components control numbers of intracellular *B*. *bronchiseptica* in the lungs.

Fewer total RB50Δ*clpV* were recovered from the lungs of C57Bl/6 mice by day 3 p.i. than total RB50, but the proportion of intracellular bacteria recovered at this timepoint were similar (Fig 6C). By day 21 p.i., RB50Δ*clpV* numbers in C57/Bl6 lungs were substantially decreased and no intracellular bacteria were observed (Fig 6D). The T6SS is therefore required for persistence in lungs of immune-competent mice, as was shown previously [29]. In the lungs of Rag1^-/-^ mice, however, 3% of total RB50Δ*clpV* were intracellular on day 3 p.i. and those numbers increased to 80% by day 21 p.i., which was substantially higher than wild type bacterial recovery in these animals (Fig 6C and 6D). Hence, loss of the T6SS enables increased recovery of intracellular *B*. *bronchiseptica* from the lungs of immunodeficient mice.

Low numbers of wild-type *B*. *bronchiseptica* were recovered from the spleens and livers of C57Bl/6 mice by day 3 p.i., none of which were intracellular in either organ (Fig 6E and 6G). Loss of *clpV* increased the proportion of intracellular bacteria in the spleens and livers of C57Bl/6 mice 3 days p.i. to 4% and 1%, respectively (Fig 6E and 6G). In Rag1^-/-^ mice RB50Δ*clpV* proportions increased to 8% in the spleen and 4% in the liver on day 3 p.i., indicating that *clpV* and adaptive immune components both limit intracellular recovery from systemic organs early during infection (Fig 6E and 6G). While no RB50Δ*clpV* were recovered from systemic organs of wild-type mice on day 21 p.i., 3% of 10^4^ CFU and 100% of 10^6^ CFU were recovered intracellularly from the spleen and liver of Rag1^-/-^ mice by day 21, respectively. This data suggests that that the T6SS prevents accumulation of intracellular *B*. *bronchiseptica* in systemic organs and that adaptive immune components are required for clearance of systemic RB50Δ*clpV*. Together these results indicate that in immunodeficient mice the T6SS prevents long-term intracellular survival suggesting that in an immunocompromised host, loss of the T6SS may aid in *B*. *bronchiseptica* persistence.

## Discussion

Many species of bacteria, including *S*. *aureus* and *P*. *aeruginosa* [50,51], utilize virulence factors to reach the bloodstream and systemic organs during infection. However, in other species bacterial factors prevent dissemination to systemic organs [52–54]. Loss of those virulence factors then causes a hypervirulent phenotype characterized by increased intracellular survival, enhanced dissemination to blood and systemic organs, and host lethality [52–54]. For example, loss of *covS* or *lgt* in *Staphylococcus aureus*, *sciS* in *S*. *enterica*, and *ccpA* in *Streptococcus pyogenes* all lead to hypervirulence and increased host lethality [47,52–54]. Although the *B*. *bronchiseptica* T6SS is required for persistence during infection of wild-type mice [29], we show that loss of this secretion system contributes to enhanced intracellular survival leading to early host death of immunodeficient mice. A large proportion of recently sequenced *B*. *bronchiseptica* strains are T6SS-negative (Fig 1), suggesting that while this secretion system plays an important role during infection [29], loss of the T6SS may also benefit *B*. *bronchiseptica* by enhancing intracellular survival. Here we showed that T6SS-deficiency is detrimental for *B*. *bronchiseptica* survival in the lungs of wild-type C57Bl/6 mice (Fig 6A and 6B). However, disruption of T6SS function is also detrimental for *B*. *bronchiseptica* in adaptive immunodeficient mice; while high numbers of RB50Δ*clpV* survived intracellularly at the site of infection, the spread of mutant to systemic organs via long-term survival in APCs contributed to host death, representing a “dead-end” for *B*. *bronchiseptica* (Figs 6 and 2). A similar phenotype for the T6SS has also been reported for *Helicobacter hepaticus*, where this secretion system plays a protective role by decreasing intracellular bacterial numbers within intestinal epithelial cells and by modulating host inflammation [55]. Since *B*. *bronchiseptica* kills its eukaryotic cellular host *via* a T6SS-mediated mechanism, a functional T6SS appears to be required for containment of the bacteria to the respiratory tract in immunocompromised mice and may potentially increase likelihood of transmission and ultimate success of this pathogen. In this work, we have not determined how the wild-type *B*. *bronchiseptica* are cleared from systemic organs of immunocompromised mice, and it will be interesting to elucidate the specific mechanism of clearance in future. Future work will determine whether loss of this secretion system correlates with intracellular recovery from clinical isolates of immunodeficient patients, and whether T6SS-deficient *B*. *bronchiseptica* strains are able to persist in the population because of their enhanced survival in an immunocompromised niche.

An adaptive immune response is required to contain *B*. *bronchiseptica* within the respiratory tract and to limit intracellular *B*. *bronchiseptica* numbers. Either B cells or T cells are sufficient to limit systemic T6SS-negative bacteria, though both are required for efficient clearance from the lungs (Bendor and Harvill, unpublished). However, a proportion of T6SS-sufficient *B*. *bronchiseptica* were recovered intracellularly from the lungs of wild-type mice during infection (Fig 6C and 6D). Intracellular localization shields these wild-type *B*. *bronchiseptica* from complement, antibodies, and other antimicrobials released during inflammation, potentially allowing them to evade these responses and emerge after resolution of the local inflammatory response. Future work will determine mechanisms of intracellular survival for these T6SS-sufficient *B*. *bronchiseptica*, including whether the wild-type bacteria persist within or repeatedly infect cells and whether they regulate expression of virulence factors and modulate host cell behavior in order to maintain their intracellular localization.

Intracellular survival of the T6SS-deficient bacteria appears to be niche specific as more intracellular bacteria were recovered in the lungs and livers than in the nasal cavities and spleens, respectively, during infection (Fig 6A–6D). This differential intracellular survival could be attributed to altered immune surveillance and response in different organs. Increased intracellular survival in the liver as compared to the spleen has also been observed with mycobacteria, where a varying immune response accounted for these differences [56]. In addition, dissimilar architecture or cellular populations of the two organs may have contributed to differences in intracellular bacterial recovery. While we used the spleen as a representative systemic organ for immune response analysis to RB50 or RB50Δ*clpV* infection in Rag1^-/-^ mice (Fig 3C), it will be interesting to determine whether a different immune response in the liver contributes to the higher proportion of surviving intracellular *B*. *bronchiseptica* during infection (Fig 6F and 6H). Also, further work identifying the cell types housing *Bordetella* in the respiratory tract and systemic organs may shed light on the niche-specific differences in intracellular survival that were reported in this study.

All three classical bordetellae (*B*. *bronchiseptica*, *B*. *pertussis*, and *B*. *parapertussis*) have been shown to be able to survive intracellularly [5,8,11,12,57]. Of the three, *B*. *bronchiseptica* is the only species predicted to have a functional T6SS [36] while the T6SS is absent from *B*. *pertussis* and degenerated in *B*. *parapertussis* [36,39]. Additionally, O antigen has been reported to mediate *B*. *parapertussis* survival in neutrophils [12] but *B*. *pertussis* lacks the O antigen locus [39], indicating that an alternative factor is likely required for intracellular survival of *B*. *pertussis*. The T3SS appears to function similarly to the T6SS by limiting intracellular survival and systemic recovery of *B*. *bronchiseptica* [58] (Bendor and Harvill, unpublished), but is probably not functional in *B*. *parapertussis* because of pseudogenes present in the locus [39]. Lastly, higher levels of *B*. *bronchiseptica* Bvg- mutants have been recovered intracellularly *in vitro* than wild-type [6], but in contrast *B*. *pertussis* appears to require Bvg function for invasion and intracellular survival in macrophages *in vitro* [59]. Thus, there seem to be different mechanisms for intracellular survival in these three closely related species, and we have identified a system in which we can dissect their varying intracellular survival strategies. This system will be useful to investigate when and how *Bordetella* species utilize an intracellular niche during infection, which will provide essential information for the design of improved vaccines and therapeutics.

## Supporting Information

S1 Fig *B*. *bronchiseptica* isolate naturally missing the Type Six Secretion System is able to colonize systemic organs of Rag1^-/-^ mice. RB50 (blue), RB50Δ*clpV* (red) and D445 (green) recovery from livers of Rag1^-/-^ mice on day 7 p.i. Grey line indicates limit of detection.
(TIFF)

S2 Fig Deletion of *clpV* increases systemic persistence in both immunodeficient and wild-type mice. RB50 (blue) and RB50Δ*clpV* (red) recovery from livers and spleens of Rag1^-/-^ and wild-type C57Bl/6 mice on days 3 (A) and 7 (B) p.i. * denotes p value <0.05.
(TIFF)

S3 Fig RB50 and RB50Δ*clpV* are both efficiently cleared when intravenously injected into mice. RB50 (blue) and RB50Δ*clpV* (red) recovery from lungs, livers, spleens, and kidneys of Rag1^-/-^ mice that had been intravenously injected and dissected on days 1 (A) and 7 (B) p.i. ND—Not Detected. The grey line indicates limit of detection.
(TIFF)

S4 Fig A *B*. *bronchiseptica* isolate naturally missing the Type Six Secretion System survives intracellularly *in vitro*. Invasion and intracellular survival of RB50Δ*clpV* (red) and a *B*. *bronchiseptica* isolate naturally missing the T6SS (D445, green) in RAW264.7 macrophages at an MOI of 100 at 1 and 24 hours post-gentamicin application. The grey line indicates limit of detection.
(TIFF)
