# Supplementary figures and images for: Type Six Secretion System of Bordetella bronchiseptica and Adaptive Immune Components Limit Intracellular Survival During Infection
Source: PLoS One. 2015 Oct 20;10(10):e0140743. doi: 10.1371/journal.pone.0140743 (PMC4618060; doi:10.1371/journal.pone.0140743)

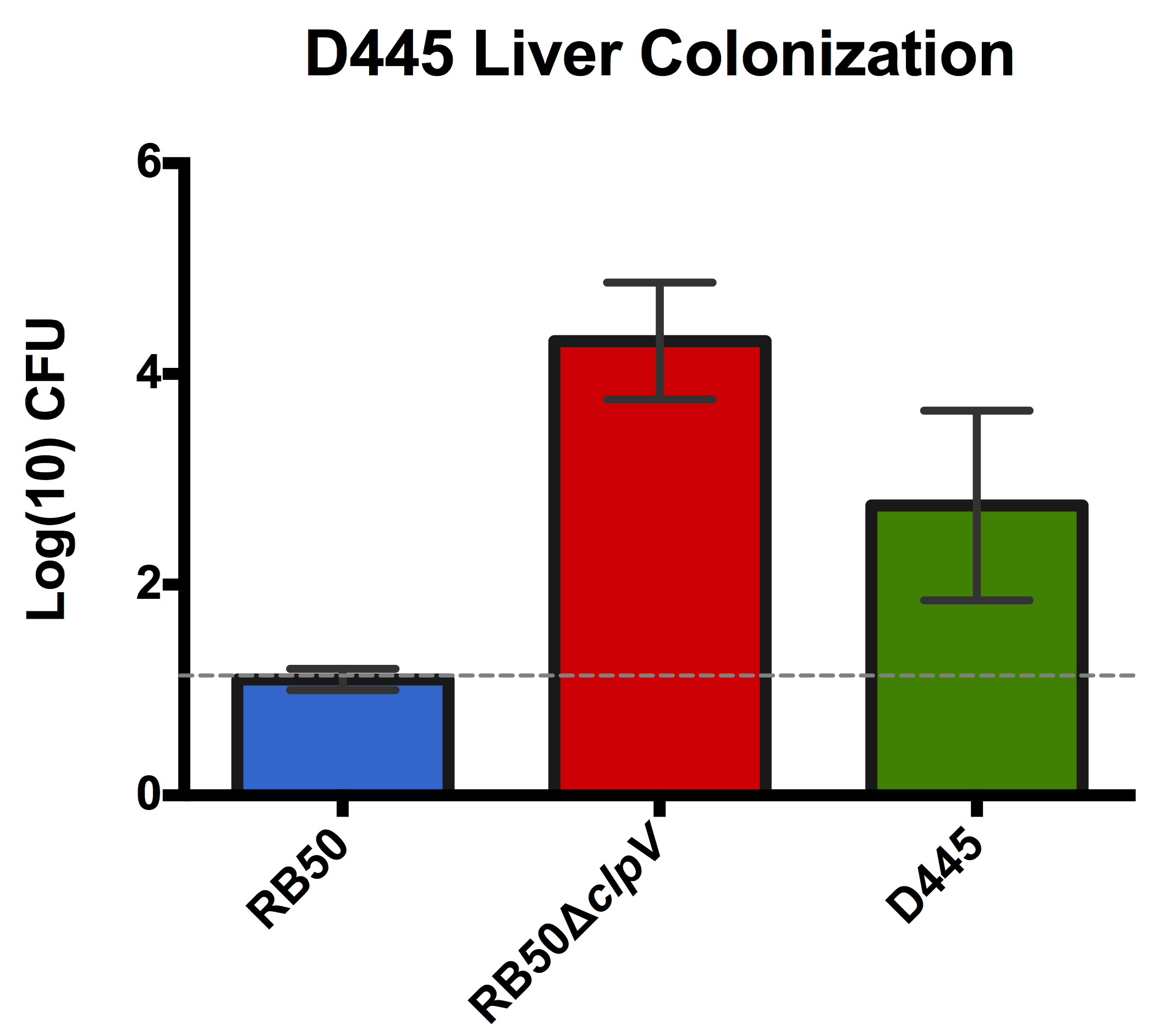

Supplement: S1 Fig — RB50 (blue), RB50ΔclpV (red) and D445 (green) recovery from livers of Rag1-/- mice on day 7 p.i. Grey line indicates limit of detection. (TIFF) [file pone.0140743.s001.tiff]

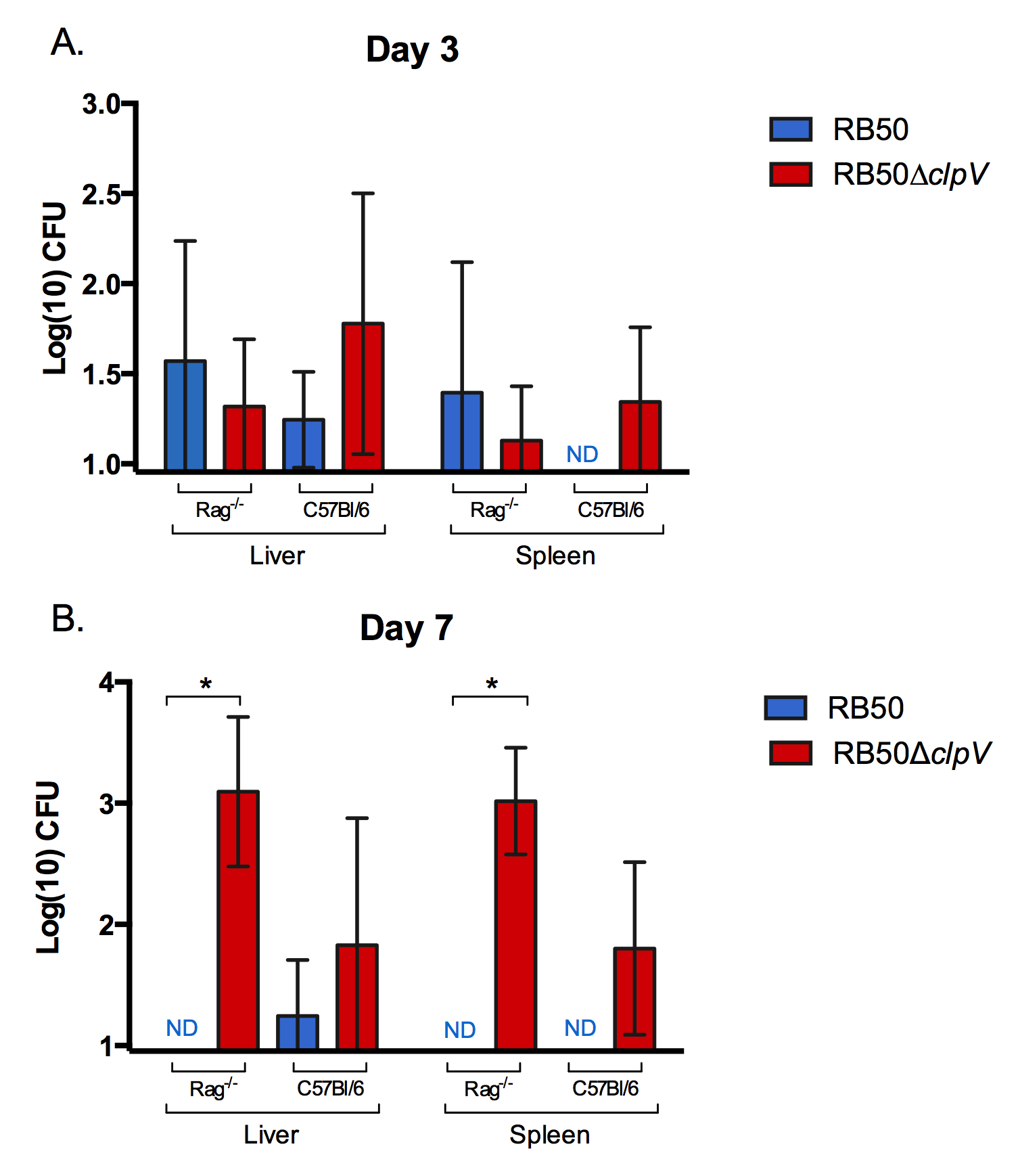

Supplement: S2 Fig — RB50 (blue) and RB50ΔclpV (red) recovery from livers and spleens of Rag1-/- and wild-type C57Bl/6 mice on days 3 (A) and 7 (B) p.i. * denotes p value <0.05. (TIFF) [file pone.0140743.s002.tiff]

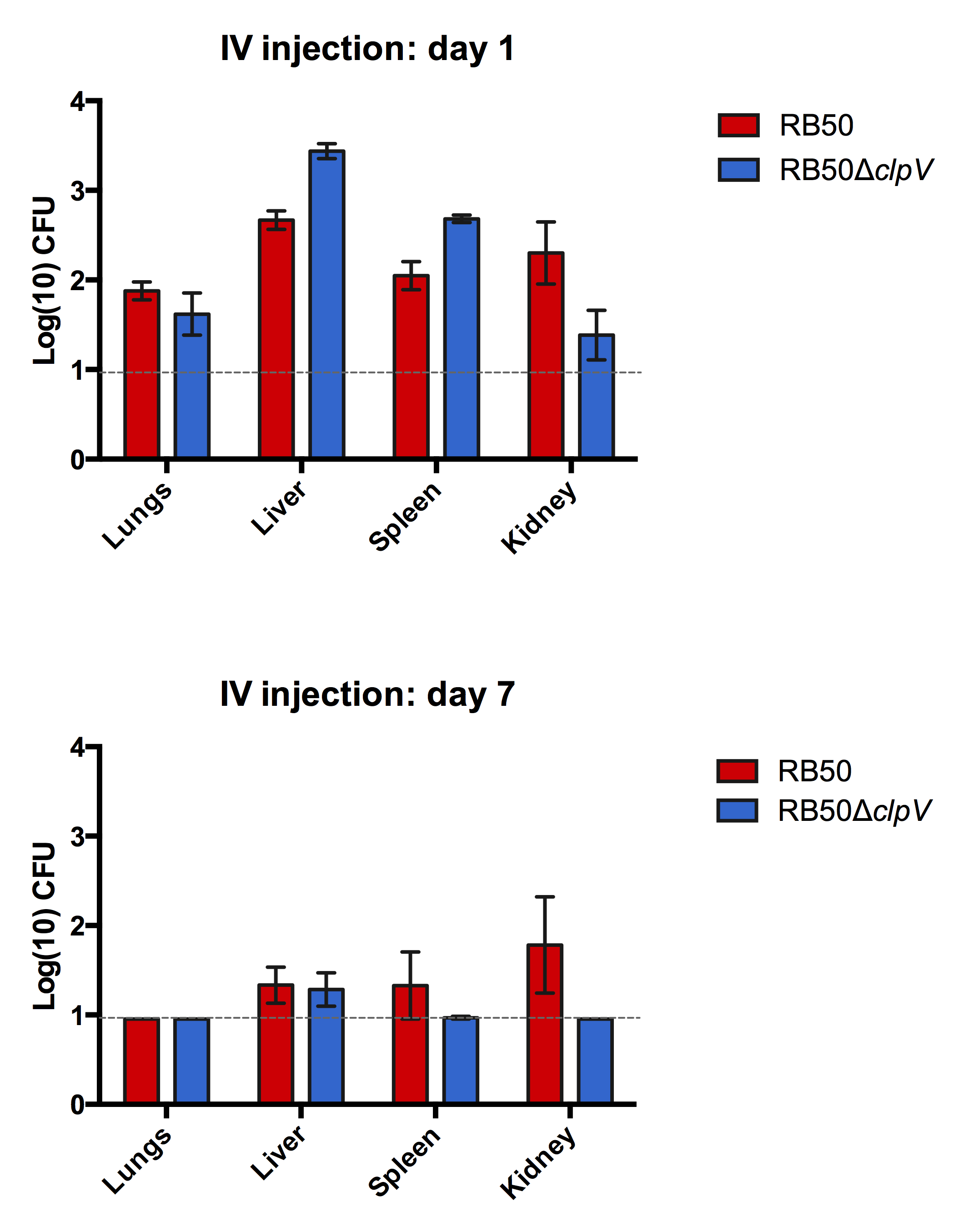

Supplement: S3 Fig — RB50 (blue) and RB50ΔclpV (red) recovery from lungs, livers, spleens, and kidneys of Rag1-/- mice that had been intravenously injected and dissected on days 1 (A) and 7 (B) p.i. ND—Not Detected. The grey line indicates limit of detection. (TIFF) [file pone.0140743.s003.tiff]

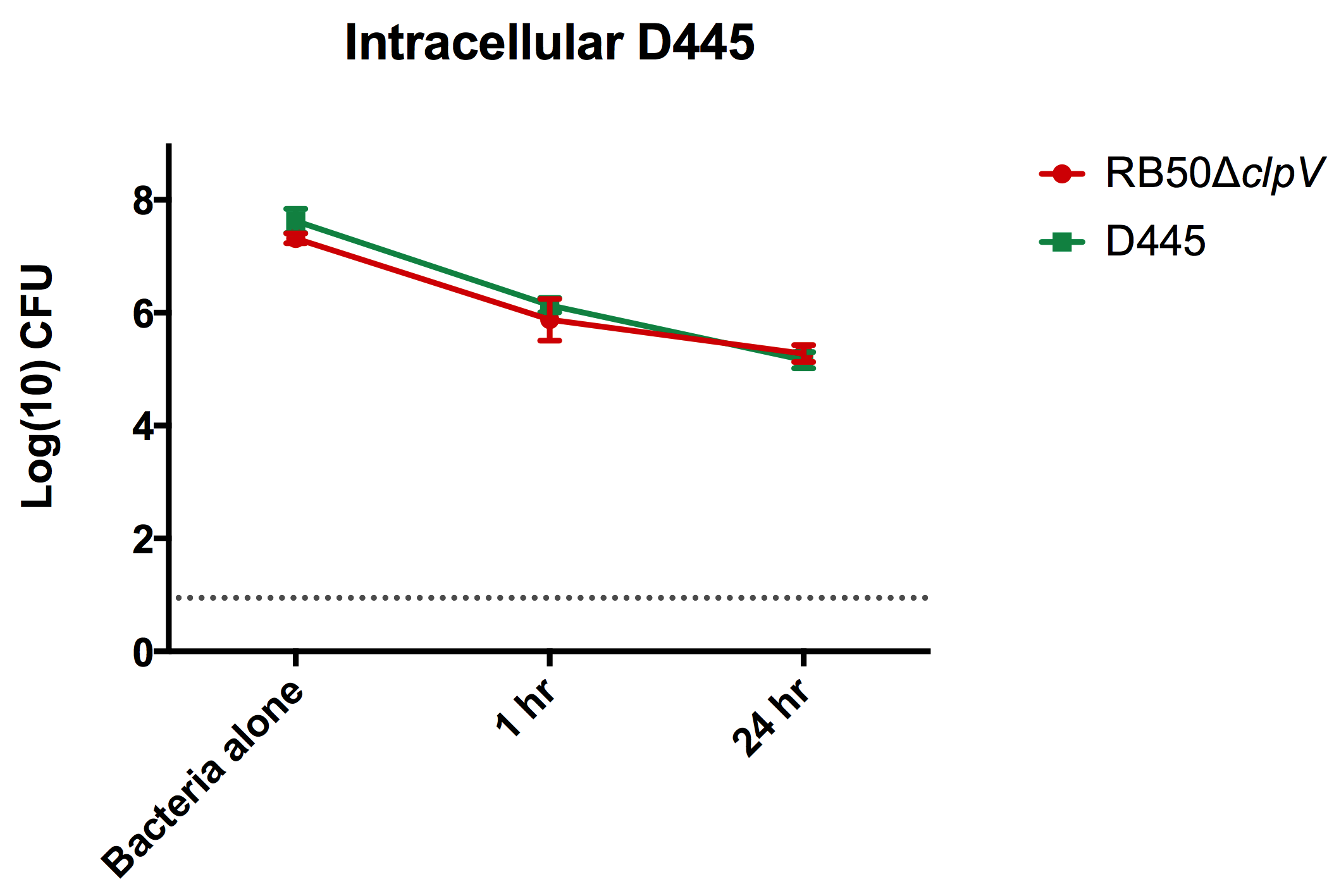

Supplement: S4 Fig — Invasion and intracellular survival of RB50ΔclpV (red) and a B. bronchiseptica isolate naturally missing the T6SS (D445, green) in RAW264.7 macrophages at an MOI of 100 at 1 and 24 hours post-gentamicin application. The grey line indicates limit of detection. (TIFF) [file pone.0140743.s004.tiff]
